# Bisphenol A in edible tissues of rams exposed to repeated low-level dietary dose by high-performance liquid chromatography with fluorescence detection

**DOI:** 10.1007/s11356-022-21154-5

**Published:** 2022-06-05

**Authors:** Vesna Cerkvenik-Flajs, Andrej Škibin, Tanja Švara, Mitja Gombač, Milan Pogačnik, Sabina Šturm

**Affiliations:** 1 Veterinary Faculty, Institute of Pathology, Wild Animals, Fish and Bees, University of Ljubljana, Gerbičeva 60, 1000 Ljubljana, Slovenia; 2 Veterinary Faculty, Clinic of Reproduction and Farm Animals, Infrastructure Centre for Sustainable Recultivation Vremščica, University of Ljubljana, Gerbičeva 60, 1000 Ljubljana, Slovenia

**Keywords:** Bisphenol A, Total bisphenol A, Sheep, Tissues, Distribution, HPLC-fluorescence, Risk assessment

## Abstract

The presented work deals with levels and distribution of bisphenol A (BPA) in the edible tissues of a large food-producing animal species. An experimental animal study included 14 young Istrian pramenka rams (*Ovis aries*), of which seven were exposed for 64 days to a low dietary dose of BPA at 25 µg/kg b.w./day, and seven served as a control group. Residue analysis of both aglycone and total BPA was performed in the muscle tissue, liver, kidney and fat tissue of the individual animals by means of enzymatic deconjugation (for total BPA), organic solvent extraction, molecularly imprinted polymer solid-phase extraction (MISPE) clean-up and high-performance liquid chromatography with fluorescence detection (HPLC-FLU). The analysis was optimized and validated for aglycone BPA in the fat tissue and for the total BPA in all tissues investigated. Edible tissues of the control group of rams generally remained BPA-free, while there were concentration differences between the control and treated groups for liver and kidney post last administration. The human health risk resulting from this study was assessed by the estimated dietary exposure in adults, which was < 0.1% related to the valid European Union Tolerable Daily Intake (TDI) value of 4 µg/kg b.w./day. However, it would be 58-fold higher than the newly proposed TDI value of 0.04 ng/kg b.w./day.

**Supplementary Information:**

The online version contains supplementary material available at 10.1007/s11356-022-21154-5.

## Introduction

Bisphenol A (BPA) is an anthropogenic compound manufactured in vast quantities around the world, with the global market for this material forecast to reach 7.1 million tons by 2027 (Report Linker 2020). It is mainly used to produce polycarbonate plastics and epoxy resins, and as such can be found in many products in daily use. Despite that the European Food Safety Authority (EFSA) set a temporary tolerable daily intake (TDI) of 4 µg/kg b.w./day in 2015 (EFSA 2015), a tremendous reduction of this value to 0.04 ng/kg b.w./day is foreseen by the recent re-evaluation of BPA due to its adverse effects on the immune system (EFSA 2021).

The endocrine-disrupting effects of BPA on animal and human health are widely researched. High-dose exposure has been connected with obesity, diabetes, cardiovascular diseases, polycystic ovarian syndrome and low sperm count (Fenichel et al. 2013; Michałowicz 2014). Low-dose exposure to BPA in animals is associated with numerous adverse neurological, carcinogenic and reproductive effects, such as predisposition of breast and prostate cells to cancer (Leranth et al. 2008; Ho et al. 2006; Murray et al. 2007; Muñoz-de-Toro et al. 2005).

**Fig. 1:**
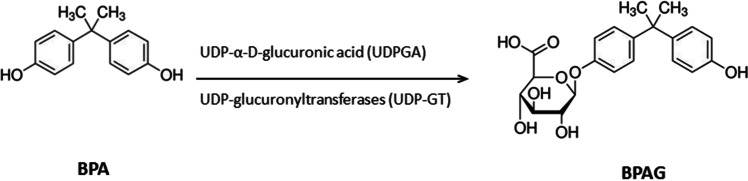
Conjugation reaction of aglycone BPA to BPAG

After oral administration, BPA in mammals undergoes a rapid conjugation, primarily with UDP-α-D-glucuronic acid (UDPGA) in the presence of UDP-glucuronyltransferases (UDP-GT) (phase II metabolism), to BPA-glucuronide (BPAG) (Fig. 1) in the intestine and liver (Inoue et al. 2004; Geens et al. 2012a). As only aglycone BPA has important estrogenic potency (Matthews et al. 2001), the formation of BPA conjugates (glucuronides, sulphates) is considered a detoxification step (Snyder et al. 2000). In humans, BPA is generally excreted via renal clearance in urine with terminal half-lives of less than 6 h (Völkel et al. 2005), while in rodents, BPA-glucuronide undergoes enterohepatic recirculation, which prolongs its elimination, primarily through faecal excretion (Pottenger et al. 2000). BPA administration at low doses (50‒125 µg/kg b.w.) induces liver damage in adult rats and liver cell death (Kazemi et al. 2017).

There is currently a lack of tissue distribution data in the literature, although the following studies have addressed this issue. Fernandez et al. (2007) tested 20 adult Spanish women to evaluate accumulation of the BPA in adipose tissue. They found aglycone BPA in 55% of the samples with a mean level of 3.16 ng/g. Sampling of human adipose tissue was very limited due to the complicated procedure (Vandenberg et al. 2010). Similarly, Geens et al. (2012b) detected aglycone BPA in human tissues (*n* = 11), where the highest mean concentrations were detected in adipose tissue (3.8 ng/g), followed by the liver (1.5 ng/g) and brain (0.91 ng/g). In an earlier study, Nunez et al. (2001) reported that repeated relatively high doses of BPA were distributed to the body tissues of rats, including adipose tissue. A US-based monitoring study found that the mean value of BPA in meat and meat products (*n* = 51) was 0.852 ng/g of fresh weight (Liao and Kannan 2013). Nevertheless, the contamination of raw meats from the environment has been significantly less researched than in the context of food processing (Siddique et al. 2021).

Analytical methods for the determination of aglycone BPA in foodstuffs as reported by Ballesteros-Gómez et al. (2009) generally covered the areas of water and beverages, infant foods, fruit and vegetables, canned foods including fish, eggs, milk, wine and spices. Turning to mammal tissue analysis, Shao et al. (2007) presented an analytical method using accelerated solvent extraction, with a subsequent clean-up step that applied amino-propyl solid-phase extraction (SPE) and liquid chromatography with tandem mass spectrometry (LC–MS/MS) in order to determine the levels of aglycone BPA in various meats, with the concentrations in the market samples ranging from 0.33 to 7.08 µg/kg. In a similar study, Deceuninck et al. (2014) used SPE HR-X, molecularly imprinted solid-phase extraction (MISPE) and gas chromatography with tandem mass spectrometry (GC–MS/MS) to examine the amount of aglycone BPA in meat and offal and assess the dietary exposure to BPA in a French population, as reported by Bemrah et al. (2014). Errico et al. (2020) developed an LC–MS/MS method for the detection of BPA in liver, visceral fat and testicular tissue of mice, where clean-up was coupled with liquid‒liquid extraction (LLE) and MISPE steps. The BPA liver toxicity and its accumulation in adipose tissue were both investigated in rats using high-performance liquid chromatography with fluorescence detection (HPLC-FLU) (Kazemi et al. 2017; Nunez et al. 2001).

Analytical methods covering both the aglycone BPA and conjugated forms in tissues are even rarer, following the principles needed to determine human metabolism and kinetics and provide biomonitoring in biological fluids (Nicolucci et al. 2017), as reviewed by Andra et al. (2016). The testing method using LC-MS/MS (Twaddle et al. 2010) assesses the distribution of BPA and its metabolites into tissues of experimental rats in different developmental life periods (Doerge et al. 2011). To the best of our knowledge, the only such analytical method developed from a food safety point of view was reported by Deceuninck et al. (2019), and encompassed direct quantitative analysis of four metabolic forms of BPA and one form of BPS in foodstuffs of animal origin by ultra-high-performance liquid chromatography with tandem mass spectrometry (UHPLC-MS/MS). BPA glucuronides and sulphates are more appropriate biomarkers of BPA contamination, since they need *in vivo* metabolism (Andra et al. 2016). Contamination with the BPA metabolites is an obvious sign that the animals have received the compound for their life. Only in this case would BPA metabolites occur *postmortem*, for the difference of aglycone BPA, which can enter the food chain during food processing and/or storage. Therefore, estimation of the BPA metabolites vs. its aglycone form is helpful to evaluate the origin of and to protect against the BPA contamination.

The current study is a continuation and upgrade of the toxicokinetic and reproductive toxicity low-dose BPA study carried out in young rams (Šturm et al. 2022), and it aimed to complementarily evaluate the BPA in the consumable tissues of animals from a food-safety perspective. Siddique et al. (2021) recently emphasized the need to evaluate the concentrations of BPA, in both aglycone and metabolized forms, found in meat samples to identify the environmental contamination. To meet the goals of the current study, a reliable analytical methodology determining both aglycone and total BPA was used for the biological matrices studied, using HPLC-FLU as the instrumental approach. In addition, dietary exposure to BPA via the potential consumption of the studied tissues was estimated in relation to respective TDIs, and the related human health risk was assessed.

## Materials and methods

### Experimental animals and study design

The study was performed on 14 young Istrian pramenka rams (*Ovis aries*), settled in a sheepfold at the Infrastructure Centre for Sustainable Recultivation at Vremščica of the Veterinary Faculty of the University of Ljubljana, Slovenia. The rams were born and bred at the Centre and were 9 months old at the beginning of the experiment. They were clinically healthy, and their weight was between 34.5 and 54 kg at that time. The rams were randomly allocated to two groups, one being the control group (*n* = 7; no. of animals 1‒7) and the other being the treated group (*n* = 7; no. of animals 8‒14). The experiment lasted 64 days. Once-daily BPA (certified reference standard of ≥ 99.0% analytical purity, Sigma-Aldrich, Merck, Darmstadt, Germany, dissolved in ethanol anhydrous at 2.5 mg/mL) was administered to the treated group in feed, at a dose of 25 µg/kg b.w., while a control group received 1 mL of ethanol daily in feed with no BPA.

On the last day of the experiment, the rams received their last dose of ethanol/BPA at 6.00 a.m. and were then euthanized, one or two animals at a time. Before euthanization, the rams were premedicated with xylazine (2 mL of Xylased 5%, i/v, Chanelle Pharmaceuticals Ltd., Loughrea, Ireland) and after 7‒10 min euthanized with pentobarbital (Exagon, Richter Pharma, Wels, Austria; 2 mL/10 kg b.w.). The rams from the control group were euthanized by the following order: no. 1 at 8:10 a.m., nos. 2 and 3 at 9:00 a.m., nos. 4 and 5 at 10:25 a.m., and nos. 6 and 7 at 11:30 a.m. The rams from the treated group were euthanized as follows: nos. 8 and 9 at 8:00 a.m., nos. 10 and 11 at 8:50 a.m., nos. 12 and 13 at 9:45 a.m., and no. 14 at 10:33 a.m. Approximately 50 g of liver, kidney, meat, and fat tissue were collected during the necropsy in separate plastic BPA-free containers and quickly frozen at − 80 °C until analysis.

### Reference standards

The certified reference standard of BPA was purchased from Sigma-Aldrich (Merck, Darmstadt, Germany) in the form of a powder with 99.0% analytical purity. The stock standard solution used in the experiments was prepared at a concentration of 200 μg/mL in acetonitrile (MeCN) and then stored in a frozen condition (at − 20 °C). The working standard solutions for the calibration and fortification of the samples for aglycone BPA determination were prepared using a mixture of MeCN/H_2_O (35/65, v/v), then refrigerated (at 4–8 °C) at concentrations of ≥ 50 ng/mL. The calibration standards of < 50 ng/mL were prepared daily throughout the experiment. The working standard solutions used for fortification of the samples used to assess total BPA (with concentrations ranging from 100 to 400 ng/mL) were also prepared each day by appropriate dilution of a stock standard solution with H_2_O.

The certified reference standard of BPA β-D-glucuronide (BPAG) was obtained from Chiron AS (Trandheim, Norway) as a solution in MeCN of 95 ± 2 µg/mL analytical purity and kept frozen (at − 20 °C). The intermediate standard solution at a concentration of 10,000 ng/mL, prepared in MeCN, was also kept frozen (at − 20 °C). The working standard solutions used for fortification of the samples, from 200 to 1000 ng/mL, were prepared daily by dilution of the intermediate standard solution with H_2_O.

### Reagents and consumables

MeCN and methanol (MeOH) used were both of HPLC gradient-grade purity and obtained from J.T. Baker (Center Valley, PA, USA). The aqueous solution of enzyme ß-glucuronidase from *Helix pomatia* Type HP-2, with ≥ 100,000 U/mL (and ≤ 7500 U/mL of sulfatase activity), formic acid 98–100%, sodium acetate anhydrous and acetic acid (glacial) 100% anhydrous for analysis were all purchased from Merck (Darmstadt, Germany). The sodium acetate buffer of 1.1 M with a pH value of 5.0 was prepared by mixing aqueous 1.1 M sodium acetate and 1.1 M acetic acid in a ratio of 70:30 (v/v).

The MISPE columns used in the experiments were AFFINIMIP® SPE Bisphenols, 6 mL, with 100 mg of sorbent, as purchased from AFFINISEP (Petit Couronne, France). The centrifuge tubes (15 and 50 mL, conical, screw cap, PP) were provided by Isolab (Wertheim, Germany), and the centrifuge tubes (15 mL, conical, glass, and 30 mL, cylindrical, PP) were purchased from Brand (Wertheim, Germany). The polyolefin complex BagLight® PolySilk® 400 blender bags (175 × 300 mm) were provided by Interscience (St. Nom, France).

### Equipment

A mini chopper (Russell Hobbs, Manchester, UK) was used to homogenize the tissue samples. A Vibra AJ‒CE/AJH‒CE (± 0.001 g) electronic balance, Vibromix 403 RVI incubator shaker and Vibromix 10 vortex were purchased from Domel (Železniki, Slovenia), as was a Centric 350 centrifuge, while a Transsonic 460/H ultrasonic bath was obtained from Elma (Singen, Germany). An additional Multifuge 1 S-R centrifuge was obtained from Heraeus (Hanau, Germany), an SPE Vacuum Manifold Visiprep 24 was supplied by Sigma-Aldrich (Merck, Darmstadt, Germany) and an N-EVAP 111 evaporator was purchased from Organomation Associates (Berlin, MA, USA). The high-performance sample blender (smasher)/homogenizer MA106401:21–06-12:E was obtained from AES Chemunex (Bruz Cedex, France). A Varian ProStar HPLC system (Varian Analytical Instruments, Walnut Creek, CA, USA) was used, which was composed of a 240 model tertiary pump, a 410 model automatic injector and a 363 model fluorescence detector, and operated using Galaxie 1.7.4.5 analytical software.

### Sample extraction and clean-up

The samples of sheep tissue were examined with regard to both aglycone and total (a sum of aglycone and conjugated) BPA. Total BPA was assessed by enzymatic deconjugation of the glucuronide bond, which converted BPAG to aglycone BPA.

#### Sample extraction

Extraction of aglycone BPA from the sheep muscle tissue, liver and kidney was performed according to the method by Cerkvenik-Flajs and Šturm (2021). Briefly, an aliquot of 2.0 ± 0.005 g of a homogenized (defrosted) sample was extracted by MeCN by vortexing and ultrasonication. The organic solvent phase was evaporated under a stream of N_2_ to an aqueous residue, being dissolved using 1 mL of MeCN and 10 mL of H_2_O, before applying onto the MISPE cartridge. Regarding fat tissue, an aliquot of 2.0 ± 0.005 g of a homogenized (defrosted) sample was weighed into the blender bag and extracted with 10 mL of MeCN by the sample blender for 1 min at fast speed. The organic phase was decanted to the 15-mL PP centrifuge tube, and the bag was drained well. The sample extract was centrifuged at 2640 × g for 10 min at room temperature, and the supernatant transferred to the 50-mL PP centrifuge tube, to which 35 mL of H_2_O was added. The centrifuge tube was vortexed, ultrasonicated for 5 min and vortexed again before applying the diluted sample extract onto the MISPE cartridge.

For testing of the total BPA in all matrices investigated, an aliquot of 2.0 ± 0.005 g sample was diluted by 3 mL of 1.1 M sodium acetate buffer with pH 5.0, then ß-glucuronidase (100 µL for muscle tissue and liver, 140 µL for kidney and fat tissue) was added, and the sample was incubated at 37 ^○^C for 8 h. Total BPA was extracted from the buffered matrix by 12 mL of MeCN, by vigorous vibromixing for 2 min and ultrasonication for 13 min. After repeated vibromixing for 1 min, the sample was centrifuged at 3345 × g for 10 min at 15 ^○^C and re-extracted using 2 mL of MeCN. The combined MeCN supernatant was transferred into a 20-mL PP centrifuge tube and evaporated under an N_2_ stream at 40 ^○^C to an aqueous residue, being dissolved using 1 mL of MeCN and 10 mL of H_2_O, and optionally centrifuged at 15 ^○^C for 10 min at 3345 × g, before applying onto the MISPE cartridge.

#### Sample clean-up

MISPE of both aglycone and total BPA in all matrices examined was performed according to the method by Cerkvenik-Flajs and Šturm (2021). Briefly, the sample extract was applied onto the MISPE column, washed with 5 mL of H_2_O and 3 mL of MeCN/H_2_O (40/60, v/v) being eluted with 6 mL of MeOH. The MISPE cartridges were used multiple times. After evaporation of the MISPE eluate to dryness, the sample extract was re-dissolved in 0.8 mL of MeCN/H_2_O (35/65, v/v) (Cerkvenik-Flajs and Šturm 2021).

### HPLC analysis

A 50-µL aliquot of the final sample extract was used for the HPLC analysis. A Hypersil GOLD C18 analytical column (150 × 4.6 mm, 3 μm particle size) was employed, which was connected to a Hypersil GOLD 3μ drop-in guard cartridge (Thermo Scientific, Waltham, MA, USA). Chromatography was carried out at room temperature using the three HPLC methods. Method no. 1, which was used for testing of aglycone BPA in muscle tissue and kidney, pumped the mobile phase comprising H_2_O and MeCN at a flow rate of 1.0 mL/min. The proportion of MeCN was held for 2 min at 35% (v/v), linearly increased from 35 to 50% (v/v) at time 2–12 min, held at 50% (v/v) at time 12–20 min, reverted to 35% (v/v) at time 20–20.5 min and held at 35% (v/v) at time 20.5–21 min (Affinisep 2015). For testing of aglycone BPA in liver and total BPA in the muscle tissue, method no. 2 was used, with the last gradient ramp (50–35% of MeCN) of method no. 1 being prolonged from 20 to 25 min (Cerkvenik-Flajs and Šturm 2021). HPLC method no. 3, which was used for testing of aglycone BPA in fat tissue and total BPA in liver, kidney and fat tissue, pumped the mobile phase comprising H_2_O and MeCN: MeOH = 1: 1 (v/v) at a flow rate of 0.9 mL/min. The proportion of organic phase was held for 2 min at 35% (v/v), linearly increased from 35 to 60% (v/v) at time 2–22 min, held at 60% (v/v) at time 22–35 min, increased from 60 to 75% (v/v) at time 35–40 min, reverted to 35% (v/v) at time 40–42 min and held at 35% (v/v) at time 42–43 min (Cerkvenik-Flajs et al. 2018). All the three HPLC methods used the excitation and emission wavelengths set at 230 and 315 nm, respectively (Affinisep 2015). The BPA sample concentrations were calculated following the external standard solvent calibration approach. In this process, the BPA concentration measured in the reagent blank sample, as tested within each sample batch, was subtracted from the baseline, study and spiked samples of the series, and the results were then used to calculate the serial recovery. The study sample concentrations, measured in parallel, were then corrected for the mean BPA recovery rate of the sample series.

### Quality assurance procedures

Tissue samples, particularly liver and kidney, were homogenized in a semi-frozen state and were stored until analysis in PP containers at − 80 °C. Aliquots were covered with MeCN as soon as possible at the start of analysis to avoid artifactual deconjugation.

Every study sample series consisted of a reagent blank sample, baseline sample, study samples and two recovery samples, as obtained by fortification of the baseline sample at the beginning of the procedure with aglycone BPA at a reasonable level. Baseline samples were obtained from the Ljubljana city market, as well as the Infrastructure Centre for Sustainable Recultivation Vremščica. The baseline concentrations of aglycone BPA were < 0.5 µg/kg in the muscle tissue and kidney, < 1 µg/kg in the liver and ≤ 1.2 µg/kg in the fat tissue, while the total BPA was < 2 µg/kg in the muscle tissue, kidney and liver and ≤ 2.4 µg/kg in the fat tissue. Daily calibration lines were constructed from 5 to 6 points ranging from 1 to 50 ng/mL for HPLC methods no. 1 and 2, determining aglycone BPA in the muscle tissue, liver and kidney, and total BPA in the muscle tissue, and ranging from 1 to 100 ng/mL for HPLC method no. 3, determining aglycone BPA in the fat tissue and total BPA in the liver, kidney and fat tissue.

### Method validation

As regards determination of aglycone BPA in the muscle tissue, liver and kidney, its validation has already been described in detail (Cerkvenik-Flajs and Šturm 2021). Therefore, validation of aglycone BPA determination in the fat tissue and total BPA in all matrices examined is presented here.

Linearity was evaluated by the regression and correlation parameters of the solvent standard calibration curves, with concentrations ranging from 1 to 50 ng/mL with 5‒6 points per curve. Recovery, repeatability and within-laboratory reproducibility of analysis of aglycone BPA in the fat tissue and of the total BPA in all the matrices were tested by fortification of a baseline sample with aglycone BPA and the BPAG (considering the molar mass ratio between BPAG and BPA of 1.7715), respectively. The fortification concentration levels of aglycone BPA in the fat tissue were 1, 5 and 10 µg/kg, while of the BPAG in the muscle tissue, liver, kidney and fat tissue they were 2, 10 and 20 µg/kg; 3, 15 and 30 µg/kg; 3, 25 and 50 µg/kg; and 2, 10 and 20 µg/kg, respectively. Repeatability was evaluated at the same time, while within-laboratory reproducibility was evaluated on the two different time occasions. We estimated the precision of the methods using the standard deviation (SD) and relative standard deviation (RSD) of the determined values, with the results then evaluated in line with the Horwitz coefficients (RSD_H_) (European Union 2002). The limit of detection (LOD) value was determined as the minimum detectable concentration of BPA from the matrix samples, with a signal-to-noise ratio of 3:1, while the limit of quantification (LOQ) value was estimated as the lowest BPA concentration for which the analysis proved acceptable of both repeatability in line with the Horwitz coefficients (RSD_H_) (European Union 2002) and recovery (to be at least 50%).

### Data analysis

Concentration differences between the control and treated groups of rams were tested with the independent-sample *t* test using MS Excel 2019 and were deemed significant for *p* < 0.05. All data are presented on a wet weight basis.

### Human health risk assessment

The risk to human health was assessed based on the potential daily consumer intake of the aglycone BPA residues from the present study in relation to both the still valid European Union (EU) temporary TDI value of 4 µg/kg b.w./day (EFSA 2015) and the recently proposed, considerably reduced TDI of 0.04 ng/kg b.w./day (EFSA 2021). The following data were taken into account:

- the mean aglycone BPA concentrations found in the edible tissues of the treated group of sheep (*n* = 7);
- the daily food basket, comprising 0.500 kg of meat, which for mammals comprises 0.300 kg of muscle, 0.100 kg of liver, 0.050 kg of kidney and 0.050 kg of fat (EMEA 2001);
- average adult human (consumer) body weight (mass) of 60 kg.

## Results

### Performance characteristics of BPA analysis

As regards analysis of aglycone BPA in the sheep muscle, liver and kidney tissue, relevant validation results were described in detail by Cerkvenik-Flajs and Šturm (2021). For this reason, only the validation results for the determination of aglycone BPA in the fat tissue and the total BPA in all the matrices investigated are presented here.

The deconjugation step was optimized regarding the BPA recovery, resulting from the known amount of BPAG submitted to enzymatic hydrolysis. The incubation time needed for hydrolysis was optimized and used further in the analytical process. The highest BPA recovery was achieved after 8 h of incubation of 1.1 M sodium acetate buffer solution of pH value of 5.0, fortified by a solvent standard of BPAG corresponding to 10 µg BPAG/kg in a 2-g tissue sample, although there was almost a yield plateau in the time interval from 6 to 16 h of incubation. The optimal quantity of β-glucuronidase was estimated by fortification of muscle tissue, liver and kidney with 10, 20 and 100 µg BPAG/kg, respectively. For muscle tissue and liver, optimum was achieved when using 100 µL of the enzyme (equivalent to 11,217 of enzyme units/2 g of sample) in the testing volume adding intervals of 60‒140 and 80‒140 µL, respectively, while for the kidney this volume was set at 140 µL of added enzyme (equivalent to 15,704 of enzyme units/2 g of sample), using the 80‒140-µL testing volume range. At the end of incubation, the tissue samples were decomposed to a considerable degree as a result of the efficient enzymatic activity. One hundred forty microliters of the enzyme was also used for hydrolysis of the fat tissue (2 g/sample) in order to efficiently decompose this hard matrix.

Typical HPLC chromatograms for analysis of aglycone BPA in the fat tissue and total BPA in the muscle tissue, liver, kidney and fat tissue are presented in Supplementary Figs. S1‒S5, demonstrating an appropriate chromatographic resolution and a BPA retention time of around 8.2‒8.4 and 15.3‒15.4 min for HPLC methods no. 2 and 3, respectively.

The linearity, as shown by the standard correlation coefficients, was good, as the “R-squared” values were ≥ 0.9990. The recovery and precision of the method are presented in Tables 1 and 2 and were determined on three concentrations for each BPA category/matrix combination. The recovery values of aglycone BPA in the fat tissue ranged from 69 to 86% (Table 1), while for the total BPA through BPAG fortification of the muscle tissue, liver, kidney and fat tissue they ranged from 45 to 79%, from 53 to 64%, from 56 to 62% and from 53 to 72%, respectively (Table 2). The repeatability of the measurements ranged from 9.1 to 23.7% for aglycone BPA in the fat tissue (Table 1), while for the total BPA in the muscle tissue, liver, kidney and fat tissue, it ranged from 5.5 to 38.8%, from 10.7 to 26.1%, from 2.0 to 19.7% and from 4.3 to 36.8%, respectively (Table 2). The within-laboratory reproducibility was from 9.8 to 28.4% for aglycone BPA in the fat tissue (Table 1), while for the total BPA in the muscle tissue, liver, kidney and fat tissue, it ranged from 7.0 to 37.1%, from 13.3 to 32.6%, from 5.5 to 19.9% and from 16.4 to 29.1%, respectively (Table 2). The RSD values increased inversely with the fortifying concentrations. However, they did not exceed the RSD_H_ values from the Horwitz equation (European Union 2002) at the lowest concentration levels. The estimated LOD/LOQ value for aglycone BPA in the fat tissue was 1 µg/kg (Table 1), and for the total BPA in the studied matrices it was 2 µg/kg with the mean recovery (*n* = 4–5) and RSD of 69‒98% and 10‒19%, respectively.

**Table 1:** Recovery and precision of analysis of the aglycone BPA in the fat tissue of sheep

Matrix	Fortification level (µg/kg)	Repeatability (*n* = 5)			Within-laboratory reproducibility (*n* = 10)			^a^RSD_H_ (%)
		Rec_mean_ (%)	SD (%)	RSD (%)	Rec_mean_ (%)	SD (%)	RSD (%)	
Fat tissue	1	75	17.9	23.7	72	20.5	28.4	45
	5	86	8.4	9.8	72	15.5	21.4	36
	10	71	6.5	9.1	69	6.8	9.8	32

^a^Horwitz coefficient of variation (RSD_H_) (European Union 2002)

**Table 2:** Recovery and precision of analysis of the total BPA in the sheep’s edible tissues, evaluated through BPAG fortification

Matrix	Fortification level of BPAG (µg/kg)	Repeatability (*n* = 5)			Within-laboratory reproducibility (*n* = 7–10)			^a^RSD_H_ (%)
		Rec_mean_ (%)	SD (%)	RSD (%)	Rec_mean_ (%)	SD (%)	RSD (%)	
Muscle tissue	2	79	30.6	38.8	73	27.1	37.1	41
	10	45	10.1	22.3	53	12.0	22.7	32
	20	56	3.0	5.5	54	3.7	7.0	29
Liver	3	64	16.7	26.1	54	17.6	32.6	38
	15	61	7.1	11.6	62	10.8	17.6	30
	30	53	5.7	10.7	55	7.3	13.3	27
Kidney	3	62	12.1	19.7	62	12.4	19.9	38
	25	58	3.2	5.5	61	5.7	9.3	28
	50	56	1.1	2.0	57	3.1	5.5	25
Fat tissue	2	61	22.5	36.8	72	20.9	29.1	41
	10	55	8.0	14.5	53	9.7	18.4	32
	20	65	2.8	4.3	57	9.4	16.4	29

^a^Horwitz coefficient of variation (RSD_H_) (European Union 2002)

### BPA concentrations in edible tissues of rams after multiple dietary administrations and human health risk assessment

The aglycone BPA and the total BPA concentrations in the edible tissues of the control and treated groups of rams (*n* = 7) at the end of a 2-month experiment are presented in Tables 3 and 4, respectively. The treated group received a dietary dose of 25 µg BPA/kg b.w./day for 64 days, and the last administration was 2‒4.55 h before euthanasia/sample collection.

**Table 3:** BPA concentrations found (µg/kg) in the control group of rams (n = 7)

Animal	Muscle tissue		Liver		Kidney		Fat tissue	
	Aglycone BPA	Total BPA	Aglycone BPA	Total BPA	Aglycone BPA	Total BPA	Aglycone BPA	Total BPA
K1	n.d	n.d	n.d	n.d	n.d	n.d	1.35	n.d
K2	n.d	n.d	n.d	n.d	n.d	**	n.d	3.03
K3	n.d	n.d	n.d	n.d	n.d	n.d	n.d	n.d
K4	n.d	n.d	n.d	n.d	n.d	n.d	n.d	n.d
K5	n.d	n.d	n.d	n.d	n.d	n.d	n.d	n.d
K6	n.d	n.d	n.d	n.d	n.d	n.d	1.03	n.d
K7	0.79*	n.d	n.d	n.d	n.d	2.00	2.26	2.69

*n.d.* not detected: < 0.5 µg/kg of aglycone BPA in muscle tissue and kidney, < 1 µg/kg of aglycone BPA in liver and fat tissue, < 2 µg/kg of total BPA in muscle tissue, liver, kidney, and fat tissue

^*^A semiquantitative concentration (LOD − LOQ) (Cerkvenik-Flajs and Šturm 2021)

^**^No sample available

**Table 4:** BPA concentrations found (µg/kg) in the treated group of rams (*n* = 7)

Animal	Muscle tissue		Liver		Kidney		Fat tissue	
	Aglycone BPA	Total BPA	Aglycone BPA	Total BPA	Aglycone BPA	Total BPA	Aglycone BPA	Total BPA
T8	n.d	n.d	n.d	4.68	0.74*	15.96	n.d	n.d
T9	n.d	n.d	1.23	3.44	0.50*	18.36	n.d	n.d
T10	n.d	n.d	n.d	3.07	1.12	23.96	1.27	4.49
T11	n.d	n.d	1.33	5.17	n.d	12.26	n.d	n.d
T12	n.d	n.d	1.65	5.37	1.03	12.97	n.d	n.d
T13	n.d	n.d	1.64	7.04	n.d	21.29	n.d	4.27
T14	n.d	n.d	1.21	4.12	0.71*	11.56	n.d	n.d
Mean	n.d	n.d	1.01	4.70	0.59	16.62	0.18	1.25
SD	0	0	0.71	1.34	0.45	4.78	0.48	2.14
% of aglycone vs. total BPA			21.49		3.55			

*n.d.* not detected: < 0.5 µg/kg of aglycone BPA in muscle tissue and kidney, < 1 µg/kg of aglycone BPA in liver and fat tissue, < 2 µg/kg of total BPA in muscle tissue liver, kidney, and fat tissue

^*^A semiquantitative concentration (LOD − LOQ) (Cerkvenik-Flajs and Šturm 2021)

The results demonstrate that the control group generally remained BPA-free, with some exceptions such as animal no. 7, where small concentrations of aglycone or total BPA or both aglycone BPA and total BPA were found in the muscle tissue, kidney and fat tissue. However, these concentrations were generally between LOD and LOQ values. In the fat tissue, aglycone and total BPA were found in the concentration ranges of 1–2.3 µg/kg and of 2.7–3.0 µg/kg in three and two rams, respectively (Table 3).

**Fig. 2:**
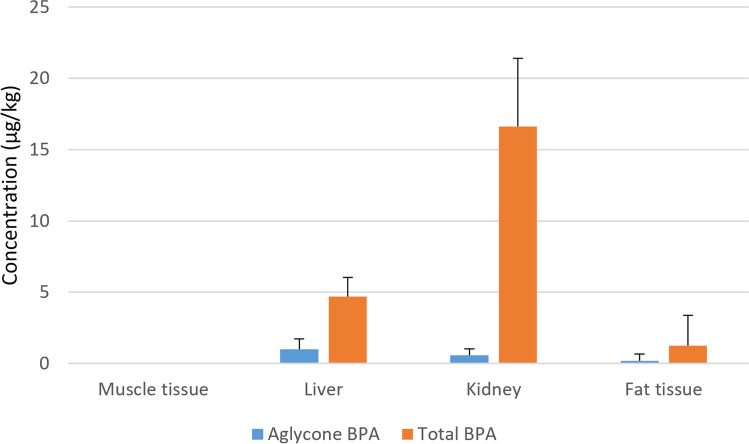
Aglycone and total BPA in the edible tissues of the treated group of rams (*n* = 7), mean ± SD concentrations found are presented

Regarding the treated group, significant BPA concentrations were found in the liver and kidney, with a minor concentration in the fat tissue and none (< LOD) in the muscle tissue (Table 4; Fig. 2). The detection frequency of aglycone BPA in both the liver and kidney was 5/7 with mean ± SD found concentrations of 1.01 ± 0.71 µg/kg and 0.59 ± 0.45 µg/kg, respectively. Aglycone BPA was found in only one sample of fat tissue out of seven animals, at a concentration between LOD and LOQ values. Total BPA was found in the liver and kidney of all animals in the treated group, with mean ± SD concentrations of 4.70 ± 1.34 µg/kg and 16.62 ± 4.78 µg/kg, respectively. Clear concentration differences between the control and treated groups of rams were confirmed for the liver and kidney by *p* <  < 0.05. The concentration ratios between the total and aglycone BPA for the liver and kidney were 5: 1 and 28: 1, respectively, and were statistically significant (*p* < 0.05). Aglycone BPA concentrations were 1.7 times higher in the liver than in the kidney, while for the total BPA these were 3.5 times higher in the kidney than in the liver. Total BPA was found in the fat tissue of two out of the seven animals, and the concentrations in these two animals were 4.5 and 4.3 µg/kg.

**Fig. 3:**
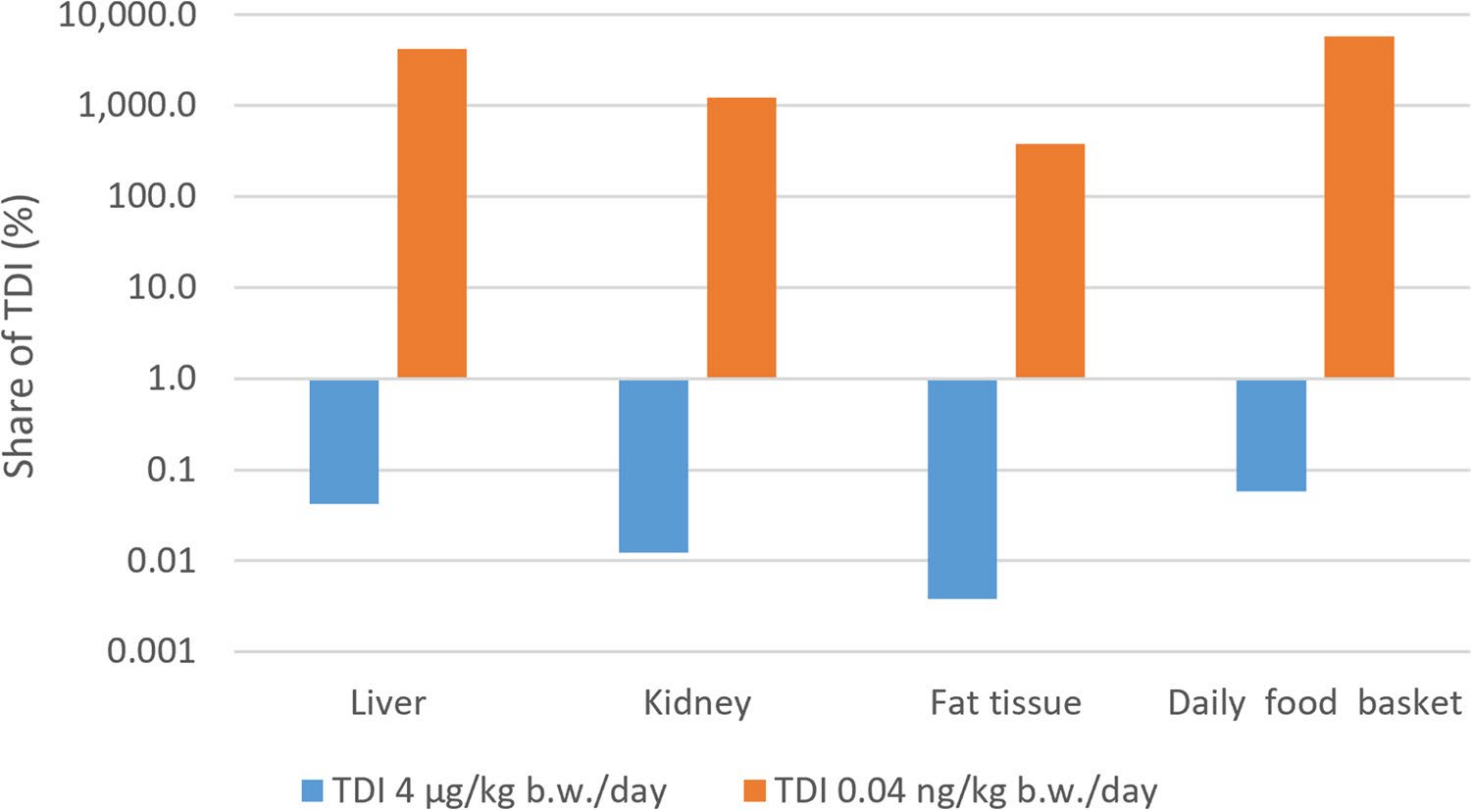
Mean shares of the potential daily consumer intake of aglycone BPA in the consumable tissues of the treated group of rams (*n* = 7) from the present study in relation to the both EU TDI values of BPA, the still valid value of 4 µg/kg b.w./day (EFSA 2015) and the recently proposed value of 0.04 ng/kg b.w./day (EFSA 2021); the daily food basket is composed of meat, incl. offal (EMEA 2001)

The mean shares of the potential daily consumer intake of the amount of aglycone BPA in the consumable tissues of the treated group of rams in relation to the established temporary TDI value in the EU of 4 µg/kg b.w./day are presented in Fig. 3, and were 0.06, 0.04, 0.01 and 0.004% for the daily food basket (comprising meat, incl. offal) (EMEA 2001), liver, kidney and fat tissue, respectively. Regarding the recently proposed, tremendously reduced TDI of 0.04 ng/kg b.w./day (EFSA 2021), these shares were 5802, 4202, 1221 and 378% for the daily food basket, liver, kidney and fat tissue, respectively (Fig. 3).

## Discussion

### Development and performance of BPA analysis

The focus of the analytical methodology presented here is in determining mainly the total BPA in the edible tissues of sheep, primarily for the purpose of assessment of BPA concentrations of multiple low-level-dose-exposed rams. Analysis of the aglycone BPA in the muscle tissue, liver and kidney has been already presented in detail by Cerkvenik-Flajs and Šturm (2021), and was complemented here by determining aglycone BPA in the fat tissue. The baseline clean-up strategy, using a single MISPE stationary phase, allied with more complex chromatographic conditions by using HPLC method no. 3, also proved to be suitable for the testing of the total BPA in the studied biological materials. New analytical optimizations were made mainly regarding the technical aspect of organic solvent extraction from the matrix of the fat tissue and the enzymatic deconjugation, for aglycone and total BPA testing, respectively.

A high-performance sample blender (smasher), an instrument usually used in microbiology, was innovatively employed in the chemical analysis for aglycone BPA extraction from fat tissue, which due to its hardness is a highly difficult matrix. Strong mechanical crushing of the sample resulted in an efficient extraction of BPA from the fat tissue matrix into the organic solvent (MeCN). As the solid–liquid extract of biological tissues contained many non-polar impurities, such as fatty acids and lipids, which can deteriorate the analytical column, and impair the chromatographic process, the use of MISPE, specifically structured to capture bisphenols, successfully facilitated the removal of interfering compounds, particularly present in the fat tissue, as emphasized by Errico et al. (2020).

The BPA standard for sample fortification of the total BPA analysis was prepared in H_2_O, with an MeCN share of ≤ 0.2% (v/v) in the sample‒buffer solution, aimed to prevent the denaturation of β-glucuronidase as in Markham et al. (2014), who demonstrated that the enzyme viability is affected at the organic solvent level of > 0.5% (v/v), particularly at lower concentrations of the analyte (< 2 ng/mL).

Optimization of the enzymatic tissue hydrolysis revealed differences with the hydrolysis method described for other matrices e.g. blood plasma and milk (Šturm et al. 2020a) and urine and faeces (Šturm et al. 2020b) regarding sample aliquot, pH of buffer, time of incubation and volume of the enzyme used. A sample mass of 2.0 g was intermediate between e.g. 0.5 mL/g used for urine/feces and 5.0 mL used for milk. Also, the incubation time of 8 h was intermediate between 4 h (blood plasma, milk, feces) and 16 h (urine), while the relative volume of the added β-glucuronidase per sample unit of 50‒70 µL/g was the largest for tissues among all matrices tested and aimed to efficiently decompose these biological materials. Nevertheless, the hydrolysis step could be omitted by direct spectroscopic measurement of BPAG and other BPA metabolites using MS (MS/MS) detection, which would also allow direct quantification of these substances in the samples (Lacroix et al. 2011; Deceuninck et al. 2019).

The obtained RSD values completely met the requirements of the Commission Decision 2002/657/EC (European Union 2002), which have been conditionally allowed, although in mid-2021 the EU set out the Commission Implementing Regulation (EU) 2021/808 (European Union 2021). It is assumed that the explanation for generally lower BPAG recovery than aglycone BPA lies either in limited matrix extraction or in partial hydrolysis. However, additional detailed studies would be needed to investigate this finding. Very few LOD/LOQ data were available in the literature for comparison with the results of our investigation. The estimated LOD/LOQ values for both aglycone and total BPA determination in the current study, found in the range of 1–2 µg/kg, were similar to the values reported by other authors (Nunez et al. 2001; Shao et al. 2007). LC–MS/MS methods were in general much more sensitive and selective, having LOD/LOQ values of one or two concentration orders of magnitude lower (Liao and Kannan 2013; Twaddle et al. 2010), which was also a consequence of the aggravated fluorescence detection capability in biological extracts at excitation/emission wavelengths below/around 300 nm.

### BPA in edible tissues of rams after multiple dietary administrations and human health risk assessment

The concentrations found in tissues after BPA treatments confirm the tissue exposure to BPA and particularly in the liver and kidney where BPA was metabolized and eliminated. These results indicate that BPA was rapidly eliminated in urine and few distributed in muscle or fat tissue. Our results are complementary with the toxicokinetic data of this study (Šturm et al. 2022), where the maximum observed total BPA blood plasma concentration (*C*_max_) after the first BPA administration was 10.93 ± 1.90 µg/L and was obtained at 1.48 ± 2.01 h (*T*_max_). As expected, the total BPA was strongly prevalent, also due to remains of urine in the kidney, in the form of BPAG. We conclude that BPA concentrations found were generally a result of the last administration, which was performed from 2.2 to 4.55 h before animals were euthanized. For comparison, the reported terminal half-lives of aglycone BPA and BPAG in sheep blood plasma following a single intravenous administration were 1.6 and 3.2 h, respectively (Gauderat et al. 2016), and the mean reported chemical half-life of the total BPA following a single subcutaneous administration was 5.3 h (Gingrich et al. 2019).

A measurable amount of BPA was detected in some of the control samples, particularly of fat tissue, probably originating from external environmental BPA contamination, as also observed by Errico et al. (2020).

Different levels of BPA concentration in the tissues of the exposed rams, particularly in fat tissue, were observed despite exposure to the equivalent dose, and were coupled primarily with variations in individual bioavailability and metabolism, allied with analytical uncertainty. Presuming BPAG as the main conjugate metabolite of BPA, the mean results for the liver of treated rams in our study correlated well with a result of 3.8 µg BPAG/kg for the liver sample (Deceuninck et al. 2019), originating from an ewe exposed to BPA at a dose of 50 µg/kg b.w./day for 105 days (Guignard et al. 2017). Distribution of BPA into tissues in our study is hardly comparable to the results of the toxicokinetic study by Doerge et al. (2011) on adult rats, due to differences in animal species and dose and route of administration. Nevertheless, aglycone BPA and total/conjugated BPA were found in the tissues tested in both studies. In Doerge et al. (2011), the adipose tissue and liver contained the highest mean found for aglycone and conjugated concentrations of 11 and 63 µg/kg, respectively. Determination of aglycone BPA was aimed at maximally preventing enzymatic deconjugation by the release of β-glucuronidase, particularly in the liver (Geens et al. 2012b). Samples were homogenized and weighed in a semi-frozen state, and extraction with organic solvent was performed under refrigerated conditions/or at a room temperature to inactivate potential enzymatic hydrolysis. The share of aglycone BPA related to total BPA in our study was 21 and 4% for the liver and kidney, respectively (Table 4).

Nowadays, incurred biological samples are highly valuable due to the 3R (replacement, reduction, refinement) principle as laid down by the Directive 2010/63/EU (European Union 2010) on the protection of animals used for scientific purposes. To the best of our knowledge, this is the first study publicly available dealing with the levels and distribution proportions of incurred aglycone and total BPA in the various edible tissues of a food-producing animal species, with rams chosen as a large animal study model. All available studies reporting incurred tissue concentrations to date have addressed the exposure to BPA from the toxicological point of view and used laboratory animals, like rats, as an experimental model (Inoue et al. 2004; Nunez et al. 2001; Doerge et al. 2011; Errico et al. 2019), while BPA tissue concentrations in food-producing animals were only reported through monitoring studies of various commercially available food items (Liao and Kannan 2013; Bemrah et al. 2014; Niu et al. 2015).

A human health risk assessment considering the potential consumer intake of aglycone BPA residues was also carried out in the present study. The calculation took into account the levels of consumption from the daily food basket comprising meat (including offal) of mammals, to protect the majority of consumers (EMEA 2001). The results were diametrically different in terms of both the EU TDI values of BPA, the still valid value of 4 µg/kg b.w./day (EFSA 2015) and the recently proposed value of 0.04 ng/kg b.w./day (EFSA 2021), with the concentration difference between them being of five orders of magnitude. While the share of the BPA content in the daily food basket in comparison to the valid TDI value was very low, at only 0.06%, it was 58-fold higher than the newly proposed TDI value. The studied matrices that contributed the most to the daily BPA intake were liver and kidney (Fig. 3). EFSA has already anticipated health concerns by comparing the new TDI with estimates of consumer both average and high exposure to BPA in the diet (EFSA 2021).

Nevertheless, the evaluated risk assessment resulting from this study reflects only a fraction of the complex total BPA human exposure when considering other items in the daily food basket, and the intake through different exposure pathways, through a wide range of consumer goods and environmental exposure from water bodies and air systems. According to Bemrah et al. (2014), products of animal origin not in cans, including meat, offal and delicatessen meats, account for 17% of the total food exposure. The fact that sheep liver and kidney are consumable, allied with the mean found total BPA elimination half-life (*t*_1/2_) in rams’ blood plasma of 7.8 ± 2.2 h (Šturm et al. 2022), of within slaughter could take place, and would contribute to the human BPA exposure, indicating health concerns. The mean BPA dietary intake of 2.32 ng/kg b.w./day in our study represented a 4.0% share of the mean daily dietary intake of bisphenols in the USA of 58.6 ng/kg b.w./day, as estimated for adults (Liao and Kannan 2013). Nevertheless, the results of a French monitoring study of BPA contamination of non-canned foodstuffs of animal origin revealed that none of the conjugated bisphenols were detected in the tested samples, indicating that the BPA contamination was not due to metabolism but occurred during food processing and from the environment (Gorecki et al. 2017).

## Conclusion

To conclude, residue chemical analysis determined both aglycone and total BPA in the muscle tissue, liver, kidney and fat tissue of sheep experimentally exposed to BPA. To the best of our knowledge, this is the first study publicly available dealing with the levels and distribution of incurred aglycone and total BPA in various edible tissues of a (large) food-producing animal species. The assessed human health risk resulting from this study is completely different when taking into account the currently valid and newly proposed, tremendously lowered EU TDI value. However, this reflects only a fraction of the complex total BPA human exposure.

## Supplementary Information

Below is the link to the electronic supplementary material.

Supplementary file1 (DOC 148 KB)

Supplementary file2 (DOC 136 KB)

Supplementary file3 (DOC 175 KB)

Supplementary file4 (DOC 181 KB)

Supplementary file5 (DOC 165 KB)

## Data Availability

All the data generated of the presented project is found in this paper.
