# Supplementary material for: Bisphenol A in edible tissues of rams exposed to repeated low-level dietary dose by high-performance liquid chromatography with fluorescence detection
Source: Environ Sci Pollut Res Int. 2022 Jun 5;29(50):76078–90. doi: 10.1007/s11356-022-21154-5 (PMC9553849; doi:10.1007/s11356-022-21154-5)
Supplement: Supplementary file 2 — Supplementary file2 (DOC 136 KB) [file 11356_2022_21154_MOESM2_ESM.doc]

A

BPA

B

C

D

**Figure** **S2.** Representative HPLC chromatograms for the determination of the total bisphenol A (BPA) in sheep muscle tissue: (A) standard 25 ng/mL; (B) muscle tissue, baseline sample; (C) muscle tissue, baseline sample fortified with 10 μg BPA/kg; (D) muscle tissue of a treated ram (T10), total BPA < 2 µg/kg.
