# Supplementary material for: Bisphenol A in edible tissues of rams exposed to repeated low-level dietary dose by high-performance liquid chromatography with fluorescence detection
Source: Environ Sci Pollut Res Int. 2022 Jun 5;29(50):76078–90. doi: 10.1007/s11356-022-21154-5 (PMC9553849; doi:10.1007/s11356-022-21154-5)
Supplement: Supplementary file 5 — Supplementary file5 (DOC 165 KB) [file 11356_2022_21154_MOESM5_ESM.doc]

A

BPA

B

C

D

**Figure** **S5.** Representative HPLC chromatograms for the determination of the total bisphenol A (BPA) in sheep fat tissue: (A) standard 10 ng/mL; (B) fat tissue, baseline sample; (C) fat tissue, baseline sample fortified with 20 μg BPA/kg; (D) fat tissue of a treated ram (T10), containing 4.5 μg total BPA/kg.
